# Hiperaldosteronismo primario en hipertensión resistente desenmascarado por hipopotasemia grave causada por una tiazida

**DOI:** 10.23938/ASSN.1174

**Published:** 2026-08-27

**Authors:** Antonio José del Rey Fernández, Ana García-Luengo Pensado, Elena Reyzabal Ereño

**Affiliations:** 1 Servicio de Salud de Castilla-La Mancha (SESCAM) Centro de Salud de Miguelturra Miguelturra, Ciudad Real España; 2 Servicio de Salud de Castilla-La Mancha (SESCAM) Hospital General Universitario de Ciudad Real Servicio de Nefrología Pediátrica Ciudad Real España

**Keywords:** Hiperaldosteronismo primario, Hipopotasemia, Hipertensión, Rabdomiólisis, Atención Primaria de Salud, Hyperaldosteronism, Hypokalemia, Hypertension, Rhabdomyolysis, Primary Health Care

## Abstract

El hiperaldosteronismo primario es la causa más frecuente de hipertensión arterial secundaria y, pese a su prevalencia, permanece infradiagnosticado en Atención Primaria.

Presentamos el caso de un varón de 71 años con hipertensión resistente y un potasio sérico en el límite inferior de la normalidad, en quien un incremento mínimo de la dosis de hidroclorotiazida (de 12,5 a 25 mg) precipitó una hipopotasemia grave (1,6 mmol/L) con alcalosis metabólica y rabdomiólisis. Un electrocardiograma realizado en consulta mostró signos de hipopotasemia y motivó la derivación urgente al hospital. El estudio etiológico reveló un cociente aldosterona/renina elevado y un nódulo suprarrenal izquierdo, estableciéndose el diagnóstico de hiperaldosteronismo primario.

El caso ilustra que una respuesta hipopotasémica desproporcionada a un reajuste mínimo de tiazida debe hacer sospechar un hiperaldosteronismo subyacente, y subraya el papel del médico de familia en su detección precoz mediante el cribado oportuno.

## Abstract (en)

Primary aldosteronism is the most common cause of secondary hypertension but remains frequently underdiagnosed in primary care.

We report a 71-year-old man with resistant hypertension and low-normal serum potassium in whom a minimal increase in hydrochlorothiazide dosage (12.5 to 25 mg) precipitated severe hypokalaemia (1.6 mmol/L), metabolic alkalosis, and rhabdomyolysis. An office electrocardiogram showed hypokalaemia-related changes, prompting urgent referral to the emergency department. Further evaluation revealed an elevated aldosterone-to-renin ratio and a left adrenal nodule, supporting the diagnosis of primary aldosteronism.

This case highlights the importance of recognizing a disproportionate hypokalaemic response to thiazide treatment as a potential indicator of underlying primary aldosteronism. It also underscores the role of family physicians in early detection through appropriate screening.

## INTRODUCCIÓN

El hiperaldosteronismo primario constituye la causa más frecuente de hipertensión arterial (HTA) secundaria, con una prevalencia estimada del 5-10% en la HTA esencial y de hasta el 20% en pacientes con HTA resistente^1^. A pesar de su elevada frecuencia, sigue siendo una entidad infradiagnosticada, especialmente en Atención Primaria, donde el cribado del cociente aldosterona/renina (A/R) en pacientes con HTA resistente o asociada a hipopotasemia continúa siendo bajo^2^.

Presentamos el caso de un paciente en el que un reajuste mínimo del tratamiento antihipertensivo desencadenó una hipopotasemia grave con repercusión sistémica que permitió alcanzar el diagnóstico de hiperaldosteronismo primario, para ilustrar la importancia del cribado adecuado y del papel del médico de familia en la detección de esta entidad.

## CASO CLÍNICO

Varón de 71 años, agricultor jubilado, que acudió a urgencias hospitalarias por crisis hipertensiva (tensión arterial 210/115 mmHg) en contexto de mal control tensional ambulatorio. Antecedentes de HTA, diabetes mellitus tipo 2 en tratamiento con antidiabéticos orales, síntomas del tracto urinario inferior y elevación de PSA (antígeno prostático específico) en seguimiento por Urología. En tratamiento crónico con dapagliflozina 10 mg/día, dutasterida/tamsulosina 0,5/0,4 mg/día, tramadol/paracetamol 37,5/325 mg a demanda, metformina/sitagliptina 1.000/50 mg/12 h, olmesartán/amlodipino/hidroclorotiazida 40/10/12,5 mg/día, solifenacina 5 mg/día y trazodona 100 mg/día (por la noche).

El estudio bioquímico realizado mostraba parámetros dentro de la normalidad, con niveles de potasio sérico en el límite inferior de la normalidad (3,5 mmol/L). Tras estabilizar la tensión arterial, el paciente fue dado de alta.

En la revisión posterior, su médico de familia intensificó modestamente el tratamiento antihipertensivo incrementando únicamente la dosis de hidroclorotiazida de 12,5 a 25 mg, manteniendo el resto de la combinación sin cambios.

Aproximadamente un mes después, el paciente reconsultó en su centro de salud por astenia progresiva, debilidad muscular generalizada de cinco días de evolución, pérdida ponderal de unos 5 kg en los meses previos, hipersomnolencia diurna y nocturna y sensación de inestabilidad. Su médico de familia le realizó un electrocardiograma en consulta que mostró signos sugestivos de hipopotasemia (descenso del segmento ST, aplanamiento de onda T y aparición de onda U), y derivó al paciente de forma urgente al servicio de urgencias hospitalarias.

En la analítica realizada en urgencias destacan hipopotasemia grave (1,6 mmol/L de potasio), alcalosis metabólica (pH: 7,53, HCO_3_¯: 45 mmol/L) y rabdomiólisis (creatina quinasa, CPK: 1.924 UI/L). Se inició reposición intravenosa de cloruro potásico por vía periférica (10 mEq/h) en observación, sin respuesta adecuada, por lo que el paciente ingresó en el servicio de Nefrología.

En Nefrología se le canalizó una vía central femoral para perfundir 20 mEq/h de cloruro potásico, con evolución progresivamente favorable. Durante el ingreso se completó el estudio etiológico: aldosterona sérica 41,1 ng/dL con renina suprimida y cociente A/R elevado, excreción fraccional de potasio del 16,6 % y gradiente transtubular de potasio (GTTK) de 25, hallazgos compatibles con pérdida renal de potasio mediada por exceso de mineralocorticoide. La tomografía computarizada abdominal con contraste evidenció un nódulo de 11 mm en glándula suprarrenal izquierda con lavado absoluto del 82,9 % y relativo del 51,5 %, hallazgos compatibles con adenoma suprarrenal frente a foco de hiperplasia nodular^3^. Con el conjunto de datos clínicos, bioquímicos y radiológicos se estableció el diagnóstico de hiperaldosteronismo primario. Al alta se sustituyó la combinación con hidroclorotiazida por valsartán/amlodipino 160/10 mg/día y se inició tratamiento con un antagonista del receptor mineralocorticoide (espironolactona 100 mg/día).

En el seguimiento ambulatorio posterior, el paciente presentó potasio sérico normalizado (4,0 mmol/L) y tensión arterial controlada, manteniéndose en seguimiento conjunto por Nefrología y Atención Primaria para valorar tratamiento definitivo (adrenalectomía o antagonistas del receptor mineralocorticoide).

## DISCUSIÓN

Este caso ilustra una situación clínica relevante para el médico de familia: un paciente con HTA resistente (tensión arterial no controlada a pesar del tratamiento con tres fármacos antihipertensivos a dosis adecuadas, incluido un diurético)^4^ y un potasio sérico previo en el límite inferior de la normalidad, en quien un incremento mínimo de la dosis de tiazida (12,5 a 25 mg) -reajuste habitualmente considerado de bajo riesgo en la práctica clínica diaria- precipitó una hipopotasemia grave con rabdomiólisis y alcalosis metabólica. La magnitud y rapidez de la respuesta hipopotasémica fue desproporcionada para la modificación terapéutica realizada y constituye, por sí misma, una señal de alarma que orienta hacia la presencia de un hiperaldosteronismo subyacente^5^.

Antes del episodio agudo, en nuestro paciente concurrían al menos dos criterios clásicos de cribado (HTA resistente y potasio basal en el límite bajo) que pasaron inadvertidos. Las guías actuales han ampliado las indicaciones del cociente A/R hasta proponer su determinación en todo paciente hipertenso^1^^,^^4^, precisamente porque el hiperaldosteronismo primario es frecuente, está infradiagnosticado y confiere un riesgo cardiovascular (fibrilación auricular, ictus, infarto de miocardio e insuficiencia cardíaca) superior al de la HTA esencial con similar grado de control tensional^6^^,^^7^. La accesibilidad y la continuidad asistencial propias del primer nivel hacen del médico de familia el profesional idóneo para detectar estos perfiles e iniciar el cribado mediante una simple determinación analítica ambulatoria.

Conviene recordar que el cociente A/R es únicamente una prueba de cribado: un resultado positivo requiere confirmación bioquímica y una posterior subtipificación (adenoma unilateral frente a hiperplasia bilateral) para decidir el tratamiento definitivo, ya sea adrenalectomía o antagonistas del receptor mineralocorticoide^1^, proceso aún en curso en nuestro paciente. Además, los diuréticos y los antagonistas mineralocorticoideos deben suspenderse al menos cuatro semanas antes de la extracción para no interferir en la interpretación del cociente^1^, un aspecto especialmente pertinente cuando el cribado se solicita desde Atención Primaria.

La rabdomiólisis hipopotasémica es una complicación infrecuente pero descrita en la hipopotasemia grave (niveles de potasio inferiores a 2,5 mmol/L), debida a la alteración del flujo sanguíneo muscular y al fallo en la generación del potencial de membrana que conduce a necrosis miocitaria^8^. Ante una hipopotasemia con HTA y alcalosis metabólica como la de nuestro paciente, el diagnóstico diferencial incluye, además del hiperaldosteronismo primario, el síndrome de Cushing, la estenosis de la arteria renal, el síndrome de Liddle y el consumo de regaliz (Tabla 1)^9^. La medición del cociente aldosterona/renina constituye la prueba de cribado inicial recomendada en pacientes con sospecha clínica, ya que permite diferenciar entre causas dependientes (renina suprimida, aldosterona elevada) e independientes de aldosterona (renina y aldosterona suprimidas).

**Tabla 1:** Diagnóstico diferencial de la hipopotasemia con hipertensión arterial y alcalosis metabólica

Causa	Renina	Aldosterona
Hiperaldosteronismo primario (adenoma o hiperplasia)	Suprimida	Elevada
Hiperaldosteronismo secundario (estenosis de arteria renal, HTA renovascular, HTA maligna)	Elevada	Elevada
Síndrome de Cushing	Suprimida o normal	Normal o baja
Síndrome de Liddle (pseudohiperaldosteronismo)	Suprimida	Suprimida
Consumo de regaliz / carbenoxolona	Suprimida	Suprimida
Hiperplasia suprarrenal congénita (déficit de 11β-hidroxilasa o de 17α-hidroxilasa)	Suprimida	Variable

HTA: hipertensión arterial.

En nuestro paciente, el electrocardiograma realizado en la propia consulta de Atención Primaria fue determinante para orientar el diagnóstico. La hipopotasemia produce alteraciones electrocardiográficas características -descenso del segmento ST, aplanamiento de la onda T y aparición de la onda U- y, en casos graves, puede predisponer a arritmias potencialmente mortales^10^. El reconocimiento de estos signos ante un paciente con debilidad muscular permitió sospechar el trastorno electrolítico y derivarlo de forma urgente antes incluso de disponer de la analítica, lo que ilustra la utilidad de una herramienta sencilla y accesible en el primer nivel asistencial.

En conclusión, el caso refuerza tres mensajes prácticos para el médico de familia: (1) un potasio en el límite inferior de la normalidad en un paciente con HTA resistente debe motivar el cribado del hiperaldosteronismo primario antes de añadir o intensificar fármacos kaliuréticos; (2) una respuesta hipopotasémica desproporcionada a un reajuste mínimo de tiazida obliga a sospechar hiperaldosteronismo primario subyacente y a derivar para estudio etiológico; y (3) la realización de un electrocardiograma en consulta ante un paciente con sintomatología compatible con trastorno electrolítico permite el diagnóstico precoz y la derivación oportuna, papel clave que el médico de familia puede desempeñar en virtud de su accesibilidad y conocimiento longitudinal del paciente.

## Data Availability

Los datos que sustentan este caso clínico están disponibles, previa solicitud razonada, a través de la persona autora de correspondencia. Por motivos de confidencialidad del paciente no se han depositado en un repositorio público.
